# Meteorological data source comparison—a case study in geospatial modeling of potential environmental exposure to abandoned uranium mine sites in the Navajo Nation

**DOI:** 10.1007/s10661-023-11283-w

**Published:** 2023-06-12

**Authors:** Christopher Girlamo, Yan Lin, Joseph Hoover, Daniel Beene, Theodros Woldeyohannes, Zhuoming Liu, Matthew J. Campen, Debra MacKenzie, Johnnye Lewis

**Affiliations:** 1 Department of Geography and Environmental Studies, UNM Center for the Advancement of Spatial Informatics Research and Education (ASPIRE), University of New Mexico, Albuquerque, NM 87131 USA; 2 Department of Environmental Science, University of Arizona, Tucson, AZ 85721 USA; 3 College of Pharmacy, Community Environmental Health Program, University of New Mexico Health Sciences Center, Albuquerque, NM 87131 USA; 4 Department of Computer Science, University of New Mexico, Albuquerque, NM 87131 USA; 5 Department of Pharmaceutical Sciences, College of Pharmacy, University of New Mexico, Albuquerque, NM USA

**Keywords:** Meteorological, Navajo nation, Particulate matter, Spatial analysis and modeling, Abandoned uranium mines, GIS multi-criteria decision analysis, Radom forest

## Abstract

Meteorological (MET) data is a crucial input for environmental exposure models. While modeling exposure potential using geospatial technology is a common practice, existing studies infrequently evaluate the impact of input MET data on the level of uncertainty on output results. The objective of this study is to determine the effect of various MET data sources on the potential exposure susceptibility predictions. Three sources of wind data are compared: The North American Regional Reanalysis (NARR) database, meteorological aerodrome reports (METARs) from regional airports, and data from local MET weather stations. These data sources are used as inputs into a machine learning (ML) driven GIS Multi-Criteria Decision Analysis (GIS-MCDA) geospatial model to predict potential exposure to abandoned uranium mine sites in the Navajo Nation. Results indicate significant variations in results derived from different wind data sources. After validating the results from each source using the National Uranium Resource Evaluation (NURE) database in a geographically weighted regression (GWR), METARs data combined with the local MET weather station data showed the highest accuracy, with an average *R*^2^ of 0.74. We conclude that local direct measurement-based data (METARs and MET data) produce a more accurate prediction than the other sources evaluated in the study. This study has the potential to inform future data collection methods, leading to more accurate predictions and better-informed policy decisions surrounding environmental exposure susceptibility and risk assessment.

## Introduction 

Exposure to pesticides, hazardous chemicals, and respirable particulate matter (PM)_2.5_ (Sharma et al., 2020) has been associated with a host of negative human health outcomes (Fu & Xi, 2020). In particular, the trace metal component of PM_2.5_ from abandoned uranium mines (AUMs) has previously been linked to cardiopulmonary toxicity (Zychowski et al., 2018). While the majority of environmental justice (Northridge et al., 2003) and health (Wellenius et al., 2006) exposure research emphasizes populations in urban areas, there remains a dearth of literature addressing exposure and health relationships in rural communities (Hendryx et al., 2010). There is also increasing recognition of the extent of contaminant exposure and human health consequences among Indigenous communities in the western USA (Hoover et al., 2019; Lewis et al., 2017).

Abandoned and inactive hard rock mines in or near Indigenous and rural communities are a critical potential source of exposure to environmental chemicals (Lewis et al., 2017). More than 160,000 abandoned hard rock mines, including over 4000 uranium mines, are located in the Western USA, which is where the majority of the Indigenous peoples in the USA reside (Hoover et al., 2019; Lewis et al., 2017). Contaminants from the waste piles and abandoned mines are dispersed through the air, water, and soil, creating a legacy of chronic community exposure. For example, the US Environmental Protection Agency stated that contaminants found in abandoned mine waste have contaminated headwaters areas in 40% of the watersheds in the western USA (USEPA, 2000), illustrating the geographic scope of this challenge.

Geospatial modeling approaches are used for environmental exposure and risk assessment to identify and quantify potential exposure and risk in a particular geographic area, including but not limited to GIS-based modeling (Malczewski, 2006; Nuckols et al., 2004), spatial statistical modeling (e.g., spatial temporal modeling) (Elliott & Wartenberg, 2004), air dispersion modeling (e.g., AERMOD) (Calder, 2008; Hadlocon et al., 2015; Holmes & Morawska, 2006; US EPA, 2016a), and environmental fate modeling (Falakdin et al., 2022). MET data (including but not limited to temperature, dew point, wind direction, wind speed, humidity, cloud cover, precipitation) are crucial inputs for geospatial modeling efforts for two major reasons: (1) environmental contaminant transport and exposure processes are highly dependent on meteorological conditions (e.g., the impact of wind on air dispersion of contaminants) (Shi et al., 2017; S. Hu et al., 2020); and (2) MET data are inherently geospatial data (Wel and Frans. , 2005) containing sophisticated spatial and temporal scales to support geospatial modeling (US EPA, 2016a).

GIS-Multi Criteria Decision Analysis (GIS-MCDA) is one geospatial modeling technique suited for applications that include MET data. Besides environmental exposure and risk assessment study, application of this MCDA framework is wide ranging (Malczewski, 2006) including remedial site evaluations (Chen et al., 2010; F. Li et al., 2018)), land use suitability (Chen et al., 2010; Chang et al., 2008; Charabi & Gastli, 2011), and public health studies (Young et al., 2010). Many studies have integrated GIS-MCDA models and fuzzy set theory to address potential uncertainties in the MCDA approach (Kuo et al., 2002), which allows for modeling generalized environmental factors when highly detailed data is lacking (Kozak et al., 2008).

Predominant sources of MET data relevant for geospatial modeling include direct measurements from meteorological stations, derived gridded data products, and satellite observations. Wind speed and direction are significant variables in our model design. Therefore, the source, quality, and characteristics of meteorological data and how it is produced are critical and may influence the model output significantly. Direct measurement at MET stations primarily represents stationary locations with an array of sensors designed to record meteorological phenomenon. There is a wide range of applications of MET station data in environmental exposure studies. MET stations have been used to model environmental exposure from pollutants such as sulfur dioxide (Rogers et al., 1999), pesticides (Tao & Vidrio, 2019), and particulate matter such as PM_10_, PM_2.5_, and NO_2_ (Lei et al., 2020). MET data are based on local meteorological station or satellite observations to provide continuous estimates in both space and time, usually at a daily or weekly time scale. Other direct measurement-based MET data, such as wind related measures derived from Next-generation Weather Radar (NEXRAD), has been used for various purposes, such as PM modeling (Yu et al., 2022).

Gridded observations, also identified as reanalysis data, are commonly generated using spatial interpolation methods. Previous studies have shown although these data provide greater spatial representation, any uncertainty in the interpolation process may be propagated in further modeling applications. Gridded data products have been extensively applied in geospatial modeling efforts. For example, gridded MET data have been used in Bayesian models to determine the extent of PM_2.5_ in urban areas (Nicolis et al., 2019), in random-forest models to determine daily concentrations of PM_10_ and PM_2.5_ (Stafoggia et al., 2019), including nationwide prediction of PM_2.5_ (Yu et al., 2021), and in quantile regressions to determine the spread of fungal spores (Grinn-Gofroń et al., 2019). While there have been cases where the uncertainty in the interpolation process has proven negligible (Elaji & Ji, 2020), this remains an underdeveloped area for consideration in the model development and evaluation process.

Satellite data are valuable for spatial analysis and modeling because the data products are continuous and provide a finer temporal data scale than many gridded data products. MET data obtained from a geostationary satellite is often used in real-time weather forecasting. The temporal resolution for this data source is excellent, with data at almost any point in time. However, because of the altitude needed to attain geostationary orbit, the spatial resolution for this data source often suffers. In environmental exposure modeling studies, this data source has been used to predict the transport of PM_2.5_ (Chu et al., 2016) and estimate the spatio-temporal air temperature using machine learning (dos Santos and Schneider, 2020). Previous studies have integrated a wide variety of MET data sources in modeling efforts. As an example, gridded data, reanalysis data, and geostationary satellite data were combined for PM_2.5_ modeling (Yu et al., 2021).

The existence of various MET data products enables widespread use of these data for modeling purposes. Some studies used ground station data to model the future climate (Belcher et al., 2010; Moazami et al., 2019). Other recent studies have compared values observed from MET stations and predicted gridded data (Bandyopadhyay et al., 2018), and tested the effects of different MET data sources on community-scale epidemiology models (Colston et al., 2018). However, evaluation of the effects that different MET data sources have on the output of large scale exposure prediction models remains underdeveloped in the literature.

With limited knowledge about what effect various MET data sources have on the predictive power of geospatial models, adoption of specific MET data source is often dependent on the availability of MET data type, use cases, or modeling approaches. For example, while MET stations can provide more accurate local observations they have limited spatiotemporal coverage (Rogers et al., 1999) and are especially sparse in rural areas. This disparity exists in part due to the cost of building and maintaining MET stations as well as infrastructure needs in rural areas, such as limited satellite coverage, cell service, and a site manager available to maintain the station (Lin et al., 2020). Complex terrain can also affect the placement of MET stations, as most stations require a relatively flat location to get an accurate reading. While local or ground-based measurement MET data are preferred over the other sources as they are usually more accurate (Rzeszutek et al., 2017), there are several scenarios where this is not viable especially in rural areas where it can be difficult to implement weather stations due to lack of infrastructure, security, or funds. In these cases, researchers often turn to other MET data products to fill this gap in weather station locations (Elaji & Ji, 2020; Trubilowicz et al., 2016; Wilgan et al., 2015). However, the effect that gridded and interpolated data have on spatial models for exposure and human health studies is unclear. To account for the shortcomings of these data sources, attempts have been made to combine the three to improve both the spatial resolution and small-scale accuracy (Albers, 1995).

Using MET data sources that have either (1) inherent errors introduced by the interpolation process or (2) limitations in spatial or temporal coverage can lead to uncertainty and error in environmental exposure studies. In the scenario of adopting local observations, for rural communities, the nearest available MET station could be in the nearest large city or town. This difference in geographic location may be small (1–5 miles) or large (100–200 miles). Using MET data from large distances can have an oversized effect on the model output. In contrast, employing satellite-based or gridded reanalysis data might not be appropriate for local-scale analysis due to accuracy concerns (Rzeszutek et al., 2017). Therefore, a more developed understanding of how MET data selection impacts modeled results is critical. The purpose of this study is to implement an existing geospatial model (Lin et al., 2020) using different MET data inputs and then compare the model predictions to refine the previous model results as these results will be used in future studies in the Navajo Nation. This paper reports two sets of activities: (1) comparison of model results using different MET data sources; and (2) determination of the MET source that produces the most accurate model result.

## Data and methods

### Study area

**Fig. 1:**
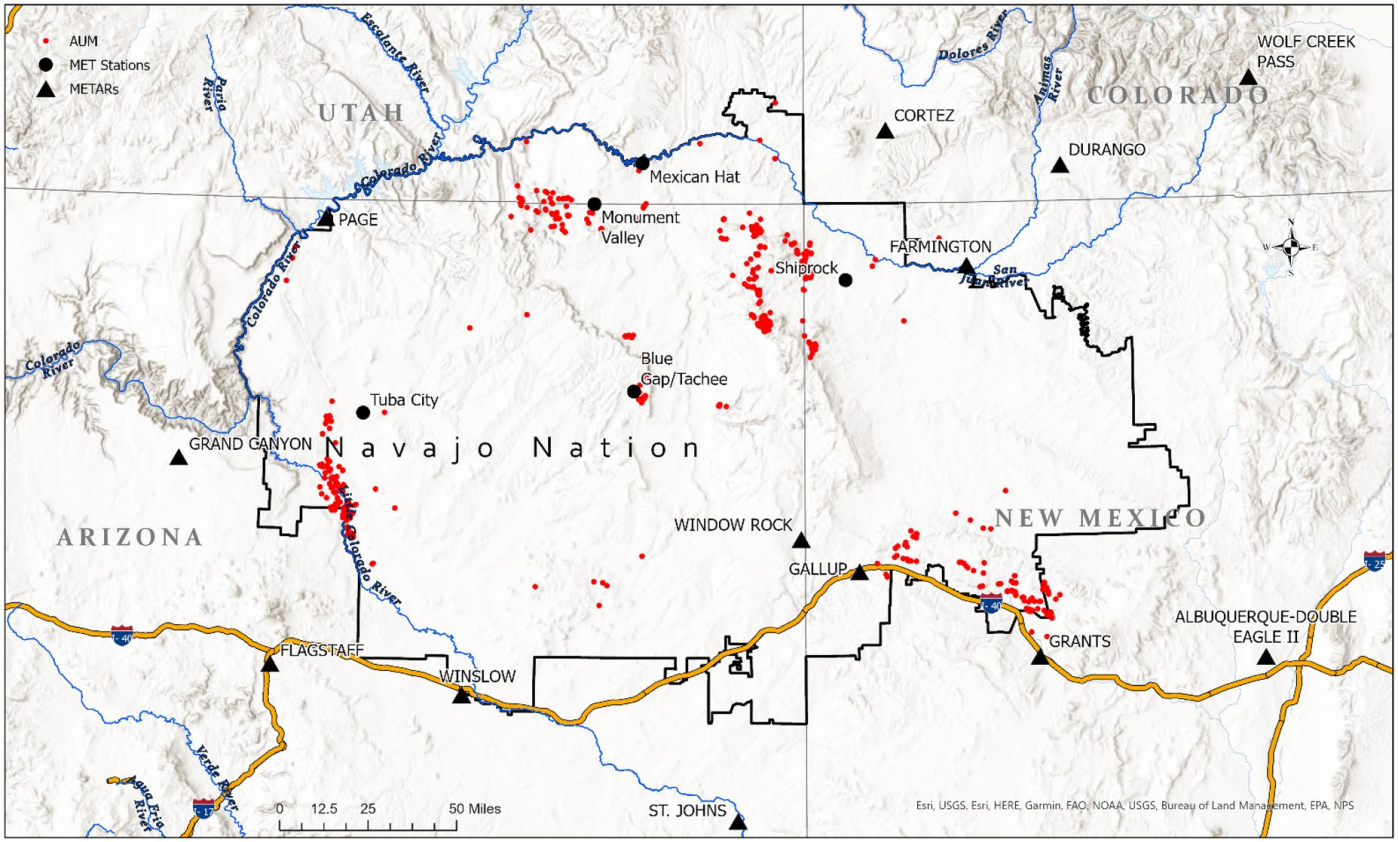
Overview map of the Navajo Nation

The study area of the present paper is the Navajo Nation, a sovereign Indigenous nation in the Southwest USA encompassing approximately 70,000 km^2^ of New Mexico, Arizona, and Utah (Fig. 1). With an estimated population of 173,000 people living on the reservation, or about 6 people per square mile, the Navajo Nation is largely rural and sparsely populated (Navajo Nation Division of Community Development, n.d.). Spanning a large swath of the Colorado Plateau, the Navajo Nation is rich in mineral and other resources. As such, there is a long history of resource extraction on Navajo lands that has left behind an intractable legacy of environmental contamination and numerous associated health risks. Waste from 523 AUMs and other mine types dispersed across the Nation is mobilized through multiple environmental pathways including surface water, groundwater, and particulate matter transported by aeolian processes. The combination of potential sources of exposure with the limited MET data available for modeling purposes suggests that this study area is a strong candidate to test the study hypothesis that uncertainties in the data source of MET data significantly influence uncertainty in model predictions and must be explicitly evaluated for their influence on results (Blake et al., 2017).

### Overall modeling framework

**Fig. 2:**
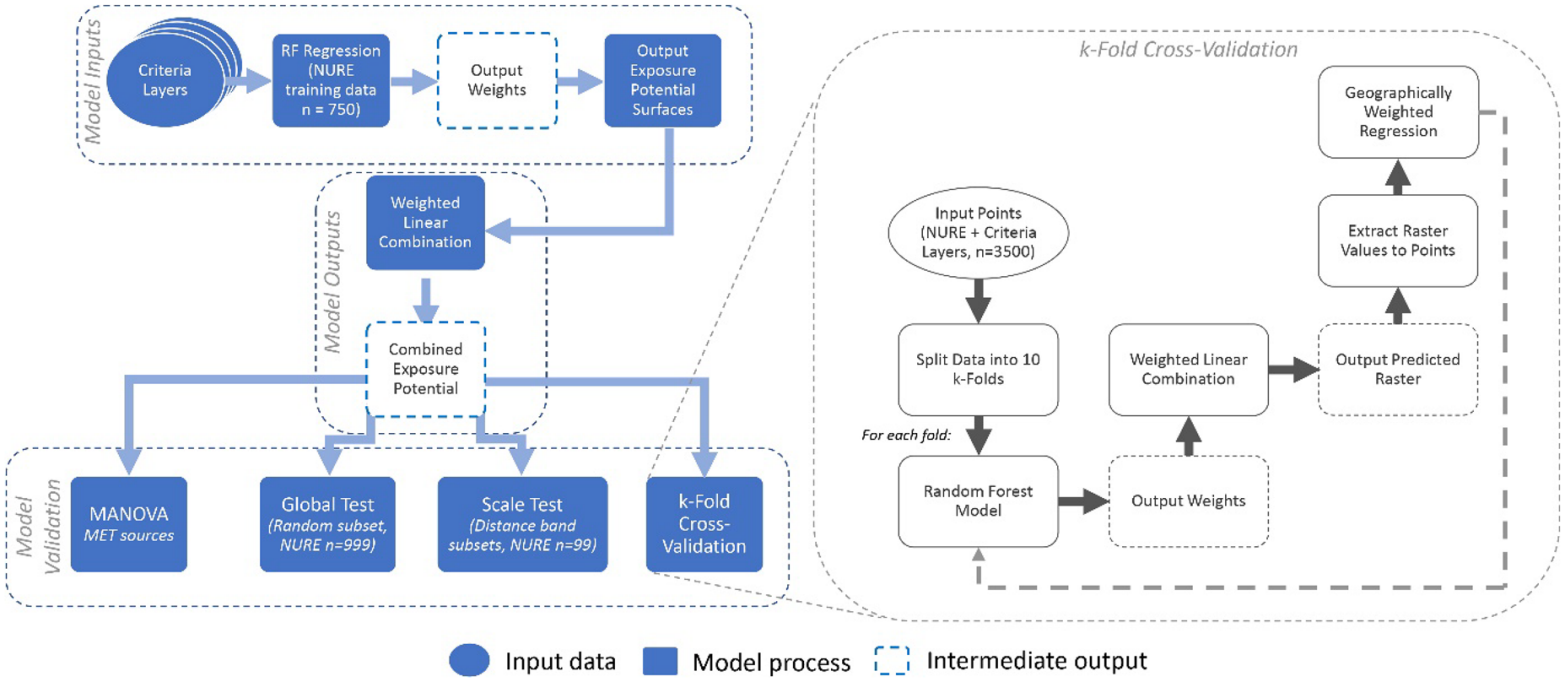
Flowchart of overall GIS-MCDA modeling approach including weight determination, and validation process with illustrating the k-fold cross validation method

Challenges of acquiring quality MET data directly affect the types of modeling frameworks that can be employed. Existing methods for modeling the dispersal of particulate contaminants, such as the American Meteorological Society/Environmental Protection Agency Regulatory Model (US EPA, 2016b) are usually based on single pathways (aerial dispersal). While such volumetric models have high accuracy in predicting the dispersal of contaminants, they require input of detailed and localized MET data and are limited to small-scale study. In contrast, GIS-based spatial models examine combined pathways and can work with different types of MET data at different scales, which makes them suitable for the purpose of the present study (Lin et al., 2020; Malczewski, 2006). Here we refined an existing GIS-MCDA model developed previously to estimate potential uranium exposure from AUMs in the Navajo Nation previously (Lin et al., 2020). We extend this model in the present work by evaluating how various MET data sources affect the model output and cross-validated results. The full model is presented in Lin et al. (2020) and is briefly summarized here (Fig. 2). Subsequent sections describe criteria layers sensitive to MET data selection, which were evaluated in the present work.

The GIS-MCDA modeling procedures for the present research (Fig. 2) included:

1. Identify and compile relevant input criteria layers.
2. Standardize each criteria layer using the fuzzy logic approach to address uncertainties in environmental risk assessment. Fuzzy membership functions applied for each criteria layer are presented in Table 1.
3. Determine criteria layer weight for weighted overlay to combine all criteria layers. Random Forest (RF) modeling approach was adopted for weight determination based on importance of each criteria layer.
4. Apply a weighted linear combination approach to combine fuzzy standardized criteria layers based on weights to produce a dimensionless exposure potential map where higher values representing a higher exposure potential to AUMs and lower value representing a lower exposure potential to AUMs. Applied a geographically weighted regression (GWR) validation method to estimate model accuracy using a separate environmental dataset—uranium concentrations in sediment and soil samples from the National Uranium Resource Evaluation (NURE) Hydrogeochemical and Stream Sediment Reconnaissance.
5. K-fold cross validation (k-fold CV) was applied. NURE data was broken into discrete training and testing subset pairs (folds). RF weight determination was performed on training sets and GWR validation on the test sets.
6. Sensitivity analysis emphasizing impact of scale of analysis. Random NURE subsets at varying scales were selected to validate the modeled results derived from each MET data input.

**Table 1:** Variables utilized in the GIS-MCDA model

ID	Criteria layer	Description	Fuzzy membership function
1	AUM proximity	The sum of inverse distance from each cell to all AUMs within 50 km, weighted by the surface area of each AUM site (Harmon et al., 2017)	MS Large
2	AUM downslope drainage	The inverse drainage path distance at each grid cell from each AUM site (illustrated in Sect. 2.4.1)	Small
3	Wind index	A sum of the difference in the angle to AUMs source and prevailing wind direction on a scale of zero to one (illustrated in Sect. 2.4.2)	Large
4	Topographic wind exposure	Angle between plane orthogonal to wind and local topography (illustrated in Sect. 2.4.3) weighted by wind speed	MS Large
5	Topographic landforms	The surrounding landforms classified into one of the following using the topographic position index (TPI) (Grinn-Gofroń et al., 2019) ridges, upper slopes, mid-slopes, lower slopes, flat land, and valleys	Small
6	Proximity to roads	The Euclidean Distance from each cell to the closest road segment	MS Small
7	Groundwater contamination	Hazard Index calculated for groundwater arsenic and uranium concentrations in more than 467 local wells; results were interpolated using inverse distance weighting interpolation	Large
8	Normalized difference vegetation index	Monthly 30-year averages of NDVI calculated using Landsat 7 and Landsat 8 imagery to represent vegetative robustness	Small

The criteria layers for the model included both non-meteorological and meteorological data sources (Table 1). Non-meteorological data sources included: (1) AUM proximity derived using AUM locations; (2) AUM downstream drainage areas; (3) wind index; (4) topographic wind exposure; (5) local topographic aspect, slope, and landforms derived from a 30-m resolution digital elevation model (DEM); (6) a roads layer for the study area provided by USEPA (US EPA, 2016); (7) a hazard index score for trace metal concentrations for 467 groundwater sources throughout the Navajo Nation (Hoover et al., 2018); and (8) a normalized vegetation index (NDVI) surface from the NASA Vegetation Index and Phenology (VIP) dataset (Didan et al., 2016). Criteria layers 1 and 5–8 are described in detail elsewhere (Lin et al., 2020) and were not altered for the present work. Criteria layers 2, 3, and 4 are described in detail in subsequent sections.

Fuzzy Large membership: monotonically increasing sigmoidal function where larger values in the input dataset have a higher degree of membership and therefore present a higher potential for contamination; Fuzzy Small membership: monotonically decreasing sigmoidal function where larger values in the input dataset have a lower degree of membership and therefore present a lower potential for contamination; Fuzzy MS Large membership: increasing sigmoidal function (defined by mean and standard deviation of input dataset) where larger values in the input dataset have a higher degree of membership and therefore present a higher potential for contamination; Fuzzy MS Small membership: decreasing sigmoidal function (defined by mean and standard deviation of input dataset) where larger values in the input dataset have a lower degree of membership and therefore present a lower potential for contamination.

### MET data

MET data from three sources (summarized in Table 2) were used to create the Wind Index and Topographic Wind Exposure criteria layers (see Table 1 above). These data sources include local airport METARs, Uranium Mill Tailings Remedial Action (UMTRA) MET stations, and gridded reanalysis data from the North American Regional Reanalysis (NARR) database.

**Table 2:** Meterological data sources employed in the present study

Sources	Category	Data type	*N*	Spatial resolution	Time period
Local Airport METARs	Direct measurements from meteorological stations	Vector	3	Undefined	1990 – 2020*
UMTRA	Direct measurements from meteorological stations	Vector	4	Undefined	2004–2021
NARR	Gridded reanalysis data based on interpolation	Raster	92	32 × 32 km	1990 – 2020
UNM METALS	Direct measurements from meteorological stations	Vector	1	Undefined	April 2021

**Sources**: Source of MET data; **Category**: type of MET data (Direct measurement or reanalysis); **Data type**: Data structure (vector or raster); **N**: number of measurements; **Spatial resolution**: the smallest spatial area with the same value (Undefined means direct measurements are not equally spaced). **Time period**: the time period that data were measured/collected; *Dependent on when each individual airport started operations and data availability; **Local Airport METARs**: airports in or near the Navajo Nation; **UMTRA:** Uranium Mill Tailings Remedial Action (UMTRA) meteorological stations; **UNM METALS**: The University of New Mexico Metals Exposure and Toxicity Assessment on Tribal Lands in the Southwest (UNM METALS) Superfund Research Program Center; **NARR**: North American Regional Reanalysis (NARR) database

#### Local airport METARs

Wind direction and speed data were collected from 13 airports in or near the Navajo Nation, among which there are only two on the reservation (Window Rock and Winslow Airport). The data was compiled and provided through the Iowa Environmental Mesonet (Herzmann, 2022). Wind direction and speed were collected on an hourly basis, and a 30-year summary of the data was used in the analysis.

#### MET stations

**Fig. 3:**
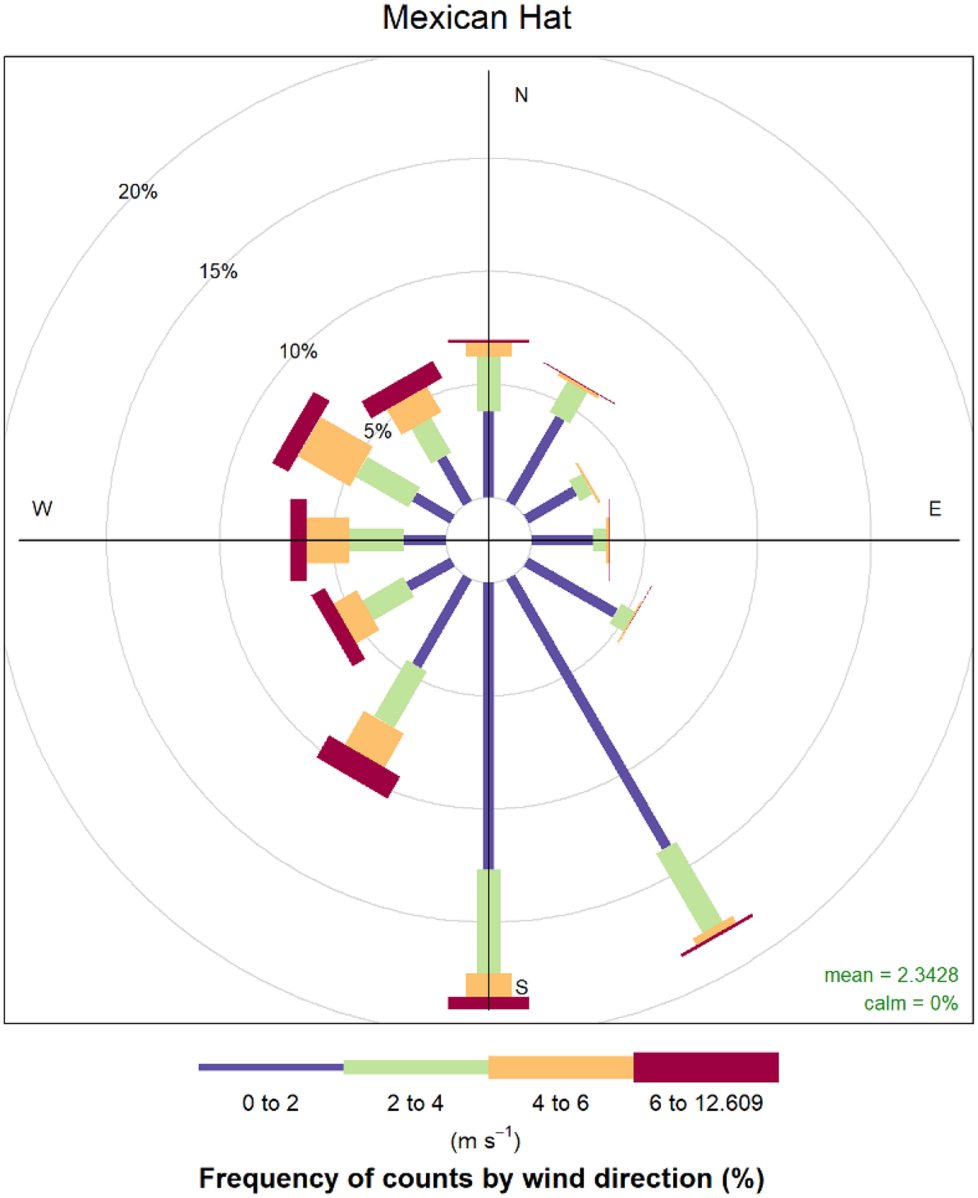
MET station wind rose example for Mexican Hat, UT

This study used four MET weather stations operated by the UMTRA program, including sites in Tuba City, AZ; Mexican Hat, UT; Monument Valley, AZ and Shiprock, NM (Office of Environmental Management, n.d.) (Fig. 1). The Monument Valley, Tuba City, and Mexican Hat stations collected data from 2017 to 2021, while the Shiprock station operated from 2004 to 2011. In addition to these stations, a temporary mobile station was also located near Blue Gap/Tachee, AZ for 2 years by the UNM METALS Superfund Research Program. The Blue Gap/Tachee station recorded wind direction and speed values every 15 min (Begay et al., 2021). For these 5 MET stations, the average wind direction was plotted in a wind rose. An example for Mexican Hat, UT is provided in Fig. 3. After plotting, the data was visually assessed, and the prevailing direction (statistical mode) for each station was used in the analysis. Wind speed was determined using the average speed for each station.

#### North American Regional Reanalysis (NARR)

The third MET data source used in this analysis was derived from the North American Regional Reanalysis (NARR) modeled dataset (NOAA, 2020). This dataset uses several input sources: radiosondes, aircraft readings, geostationary satellite cloud drifts, surface stations, and satellite radiances to model wind direction and speed across North America. It is widely used as an input for hydrological models (Trubilowicz et al., 2016), soil moisture and evapotranspiration models (Keshta & Elshorbagy, 2011), and modeling PM_2.5_ concentrations (X. Hu et al., 2013). It is because of NARR’s wide use as an input for a variety of models that it was chosen as a comparison to direct measurements. This dataset provides data hourly estimates, which was used to produce a 30-year average for equally spaced points in a 32 km $\times$ 32 km grid. For this study, the 30-year prevailing wind direction and average wind speed grid points across the Navajo Nation were used.

### Criteria layer creation

As discussed in Sect. 2.2, criteria layers 1 and 5–8 were unchanged for the present analysis. For this work the downstream drainage model (criteria layer 2) was created using a different method than our team’s previous work, and the wind index (criteria layer 3) and topographic wind exposure (criteria layer 4) were created and incrementally adjusted for the present work. The main variables in the model affected by MET data are the wind index and topographic wind exposure.

#### Downstream drainage

Pollutants from abandoned mines may be transported via surface water through ephemeral or perennial drainages. The heterogeneous spatial distribution of heavy metal concentrations in soil and sediment is influenced by surficial runoff (Hou et al., 2017; Herngren et al., 2005). Mineral ore extraction is an important anthropogenic source of elevated heavy metal concentrations in those media (Candeias et al., 2014; Z. Li et al., 2014). Previous research has demonstrated the potential of downstream drainage transport of AUMs contaminants (deLemos et al., 2009; Lameman & Terri, 2012).

Downslope drainage was characterized using a regional point-source apportionment model (Huang et al., 2015) relating downstream distance of hydrologic response units (HRUs) from mines. HRUs are unique combinations of soil type, land use and land cover, and slope that represent areal regions with similar hydrologic properties at the soil–vegetation–atmosphere interface. As a preprocessing for the downslope drainage layer, we computed a total of 8168 HRUs in the study area extent using a 30-m DEM, the 2019 USDA National Land Cover Database (NLCD), and the National Resources Conservation Service (NRCS) STATSGO2 soils database in the ArcGIS Soil–Water Assessment Tool (ArcSWAT) (Version 2012.10_4.21).

The downslope drainage criteria layer was created following the following steps:

1. The downstream drainage path from each AUM source was computed using the trace downstream tool from ArcGIS Online Ready-to-Use services, which draws a polyline drainage path from any given AUM point on land to its terminus in the ocean;
2. Because the 8163 HRU features generated using ArcSWAT were in multi-part format, meaning that multiple smaller features were grouped into single polygons with common HRU definitions, multi-part polygons were converted into 1.3 million single-part features and the centroid of each feature within 1364.39 m (the mean shape length of all single-part features) of the downstream route was snapped to the polyline;
3. The total distance along the polyline between each snapped HRU centroid and the respective polyline point source was computed using network analysis tools in ArcGIS Pro (version 2.9); and
4. The downslope drainage criteria layer was generated as the sum of all inverse distances to each AUM point source. HRU polygons which do not intersect a downstream drainage path were assigned a value of 0. Because multiple streams tend to converge into common drainages, nearly all HRU points have multiple AUMs sources and thus distance measurements.

All processes to manage HRU data and compute distance were automated with Python.

#### Wind index

The wind index was calculated using the formula we developed:

$$Wi=\sqrt{\sum_{j=1}^m(\left(\frac{1-\cos\;\left(180+\left(\theta ij-\beta\right)\right)}{2\ast Dij}\right)\ast Sv_i)}$$

$$if\frac{1-\cos\;180+(\theta if-\beta))}{2\ast Dij}>0.5$$

where $\theta$ is Euclidean direction of a receptor location from a pollutant source, $\beta$ is the prevailing wind direction of the receptor location in degrees, $S{v}_{i}$ is the scaled wind speed, and *D* is the distance between points. The wind index is a function of the relative location from each pollution source and both wind direction and speed in the surrounding geographic area.

#### Topographic wind exposure

The topographic wind exposure surface is a combination of the local terrain derived from a DEM and the wind direction. It is a combination of two planes, the orthogonal wind direction and a plane representing the local topography. The topographic wind index used in this model is based on the equation:

$$\cos\;\alpha=\cos\;(\mu)(\sin\;(\beta))+\sin\;(\mu)\cos\;(\beta)\cos\;(\delta-y)$$

where cos*α* is the angle of topographic wind exposure, *μ* is the terrain slope calculated from a DEM, *β* is the horizontal angle of wind, *δ* is the wind direction, and *γ* is the terrain aspect also calculated from a DEM (Lin et al., 2020).

#### Iterations of criteria layers pertaining to MET data

Individual MET data sources were evaluated (along and in combination) to assess the effect of each input on the model output. The Wind Index and Topographic Wind Exposure criteria layers were created using the following MET data combinations (Table 3): METARs alone; MET weather stations alone; NARR data alone; METARs and MET weather stations; NARR data and MET weather stations; NARR data and METARs; NARR data, MET weather stations, and METARs (Table 3).

**Table 3:** Meteorological data combinations

Combination ID	Combination name	Data type	*N*
1	MET weather stations	Direct measurement	5
2	METARs	Direct measurement	13
3	NARR	Reanalysis data	192
4	METARs, MET weather stations	Direct measurement	18
5	NARR, MET weather stations	Reanalysis data and direct measurement	179
6	NARR, METARs	Reanalysis data and direct measurement	155
7	NARR, MET weather stations, METARs	Reanalysis data and direct measurement	143

*N*—number of measurements

### Data integration and preprocessing

**Fig. 4:**
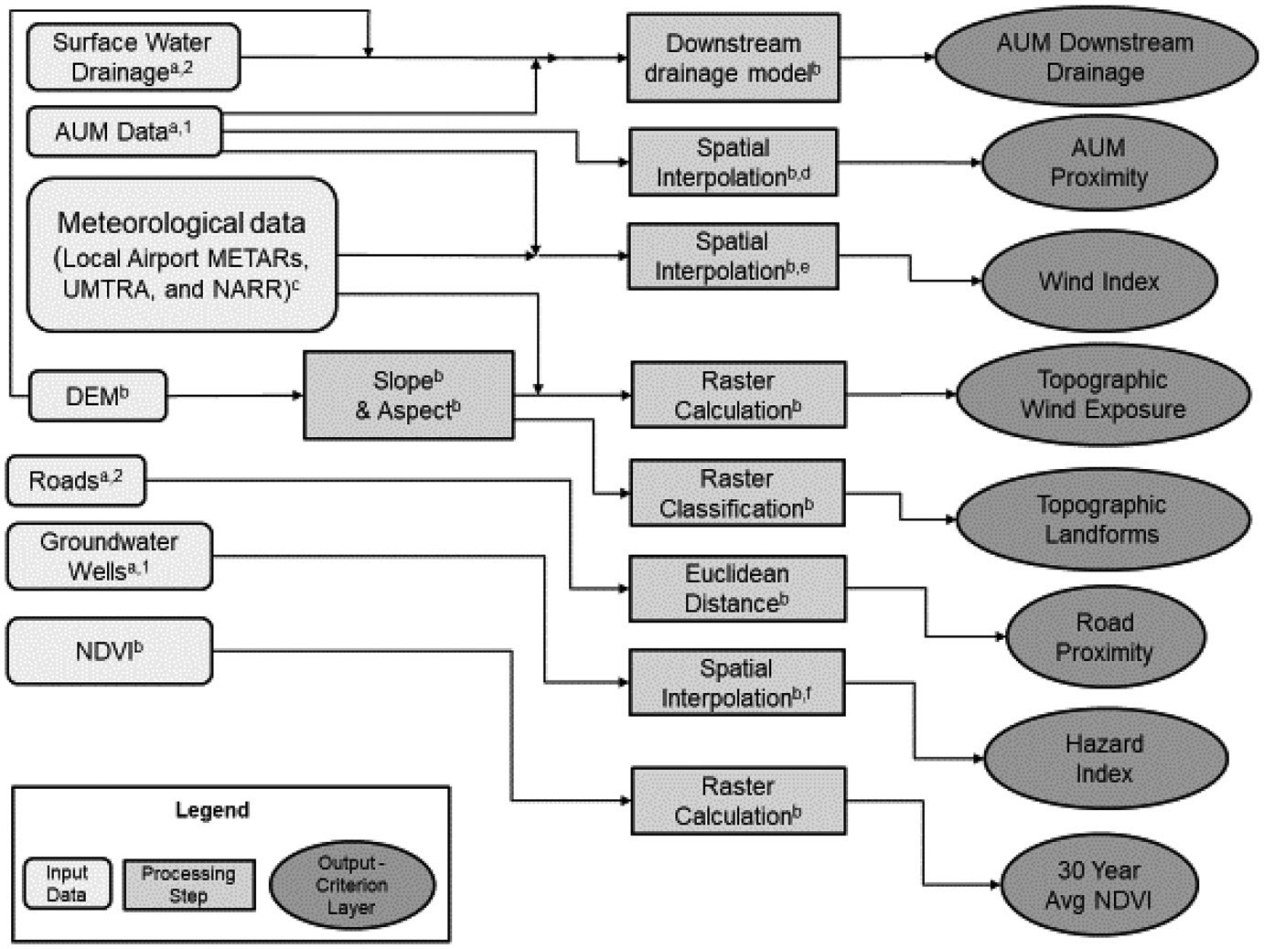
Illustration of data inputs, main processing steps, and output criteria layers (30 × 30 m resolution) employed in the present analysis. **Notes**.^1^point; ^2^line; ^a^vector spatial data format; ^b^raster spatial data format, 30 × 30 m resolution; ^c^input meteorological data including local airport METARs, Uranium Mill Tailings Remedial Action (UMTRA) MET stations, and gridded reanalysis data from the North American Regional Reanalysis (NARR); ^d^sum of inverse distance from each cell to all AUMs within 50 km, weighted by the surface area of each AUM site; ^e^sum of the difference in the angle to AUM source and prevailing wind direction on a scale of zero to one; ^f^calculated for groundwater arsenic and uranium concentrations (MCL) in more than 467 local wells; results were interpolated using inverse distance weighting interpolation; Abbreviations. AUM abandoned uranium mine, DEM digital elevation model, NDVI Normalized Difference Vegetation Index

Data integrations and preprocessing including data format and resolution can be found in Fig. 4. Data input includes AUM point feature, MET data (wind direction and speed) from different sources, DEM, DEM derived Slope and Aspect, Drainage line feature, road line feature, groundwater well point feature, and NDVI. Except for Topographic wind exposure, landforms, and NDVI directly derived from raster layers (e.g., DEM, Slope, and Aspect), other criteria layers were created from vector data input and rasterized at the same resolution for the modeling process. Detailed descriptions of how each criteria layer was generated from the data input can be found in Table 1 and Sect. 2.2 and 2.4.

As shown in Table 2, the spatial resolution of each MET data source differs. Various methods for combining and integrating data sources with varying spatial resolution can impact the results of geospatial modeling (Cotter et al., 2003). To create the above MET data combinations (Table 3), the following methodology was utilized. First, MET data layers were overlaid based on each combination. When NARR data was present in the combination (Table 3), the 4 nearest NARR points were replaced by the nearest direct measurement source. For each version, the continuous area encompassed by the Navajo Nation was classified using the nearest data points mode wind direction and mean speed value. The above process uses the closest data source in each combination for every location, assuming that the nearest data source will be appropriate in each case. A distinct Wind index and Topographic Wind Index layer was generated for each combination of MET data.

### Criteria weight determination and weighted linear combination

A criteria weight determination process can be highly subjective (Omair et al., 2021), due to a reliance on the opinions of domain experts in weight determination (Eldrandaly, 2013). In an effort to address subjectivity introduced in this process, this research applied a statistical, regression-based approach for weight determination. There are many standard regression methods for determining coefficient weights, such as multiple and multivariate linear regressions (MLR). While simpler to interpret and well-tested, linear regressions have the limitations of assuming linear relationships between independent, continuous (or coded categorical) predictor variables, and show poor performance with highly skewed data (Freedman, 2009; Schervish, 1987). Our model is highly complex, dealing with non-linear interactions between the different environmental variables that are not completely independent of each other. For example, criteria such as wind index and topographic wind exposure that both incorporate meteorological information display collinearity. The predicted exposure distributions from each criterion are highly skewed toward low predicted exposure values. While our model criteria layers are fuzzified, the landforms layer is technically categorical, in which predicted exposure values are distributed in discrete groups, as based off the original input categorical landform data.

Given the limitations of above traditional approaches, we looked into using well documented supervised ML methods, such as decision tree (DT) learning (e.g., Classification and Regression Tree (CART)) and multi decision-tree algorithms (“forests” of decision-trees, i.e., decision forests), including algorithms such as RF, to determine criterion weights for our models. RF, as developed and termed by Leo Breiman, is a multi decision-tree algorithm that utilizes a randomized “forest” of CARTs, in which each CART generates its own prediction and is input into a voting scheme to calculate final predictions. This helps to limit problems of overfitting in addition to providing better predictive power and accuracy. RF does not assume linearity, can handle continuous and categorical variables simultaneously, and is stable with complex, skewed datasets. (Breiman, 2001). Among others, RF models are now one of the most popular and commonly used algorithms by data scientists (Wu et al. 2008). As part of the regression, RF evaluates the importance of each model variable (such as criteria layers in an MCDA) through the Gini Impurity statistic (Nembrini et al., 2018).

Because of the above documented strengths and widespread use of RF, we applied RF as a regressor for weight determination of our environmental criteria. To determine an appropriate subset size for training, several subsets of the data were tested incrementally. The RF regressor model was executed using subsets of the NURE data ranging from 500 to the maximum number of points (~ 7000) at intervals of 500. The NURE HSSR program was initiated by the Department of Energy in 1973 and picked up by the USGS in 1995 to measure uranium concentration in stream sediment across the USA (USGS, 1980). The NURE database has detailed records of uranium concentrations along with several other heavy metals. The importance scores for each criterion were then plotted at each subset level. After comparing the plots, the importance scores started to become unstable at ~ 750 points, which was the subset size used for subsequent training of the RF regressor. We aimed to use the smallest-possible training set size to minimize data-overlap. Each criterion was fit to the NURE subset (*n* = 750) in the RF-regressor weight determination model. RF was implemented using Scikit Learn in a Python 3 environment.

With criteria layer weights determined, weighted linear combination was applied to all criteria layers to produce the final results of exposure potential. Because each MET data version (Sect. 2.3.2) generated a distinct wind index and topographic wind exposure layer, each produced a distinct final result layer of exposure potential to AUMs.

### Validation

#### Geographically weighted regression (GWR)

To validate the modeling results, the NURE data was regressed against the potential exposure surfaces generated using each wind version (see Table 3 for tested combinations). The validation process was conducted through fitting a geographically weighted regression (GWR) model between the modeled results and the NURE data. GWR was adopted because it is a widely employed statistical test of fit used to model spatially varying relationships (Fotheringham et al., 1998) which is appropriate given the spatial dependence of data in this study. This study quantified the match between modeled results and NURE uranium concentrations using the generated *R*^2^ values produced using the GWR method which is a measure of the regression between the explanatory (in this case the modeled potential exposure) and the dependent (NURE uranium samples) variables. The results range from 0 to 1, with 1 implying that the modeled results accounts for all the variation present in the NURE uranium concentrations, and 0 signifying the opposite. *R*^2^ was used to determine the performance of wind data in the modeling and the version of wind data input producing the highest *R*^2^ in the validation was the most accurate out of all versions of wind data. 

To determine an appropriate subset size for validation, several subsets of the data were tested incrementally. A GWR model was executed using subsets of the remaining NURE data (not used for weight determination training in Sect. 2.6) ranging from 500 to the maximum number of points at intervals of 500. The *R*^2^ results were then aggregated at each subset level and plotted. After comparing the plots, the aggregated *R*^2^ results level off at ~ 3500 points, which was the subset size used for subsequent validation and testing. We randomly selected 3500 NURE points and did the above validation. This process was repeated 999 times, to ensure that the results were not a product of chance due to the random NURE points selected.

#### K-fold cross validation

One area of validation often over-looked is over-fitting, which is a frequent issue in ML driven and/or regression based models, in which model fit can be over optimized simply by increasing sample size (Gavrilov et al., 2018). We adopted a commonly used approach to deal with potential overfitting issue due to large *N* in our study—k-fold cross validation—a method of splitting training and validation datasets into randomized (and in the case of spatial science) geographically distinct subset pairs (folds). The model is then run across the list of subsets, and outputs are assessed for stability at a given sample size (Wong & Yeh, 2020). This helps with confidence in model results, and additionally reduces the potential for training set bias caused by data leakage (Shim et al., 2021). To complete a robust k-fold validation, 10 folds (splits) were used. For each fold, RF was run on the training subset (*n* = 3500) to generate layer weights, with these weights input into the MCDA workflow. This produced a predicted exposure surface for each fold. Values from this surface were used to create a testing subset (*n* = 750) on each fold. GWR was ran on the test subsets for final validation. K-fold CV was performed using Python.

### Statistical analysis

Analysis of variance (ANOVA) was used to compare model prediction surfaces and test the null hypothesis that the mean prediction value for each prediction surface was not different. A random set of 500 values was selected because of the large number of values in the predicted exposure potential surface. The data was log-transformed (base 2) for comparison to account for significant right-skewness. Under these conditions, the distribution of the data looks relatively normal, with an average Pearson mode skewness of 0.59.

### Scale variability analysis

This study also investigated how the model results varied at different scales. To do this, 99 random points within the study area were selected as test sites. From each test site, the GWR process was performed at distances of 25, 50, 75, 100, 125, 150, and 175 km from each test site for each result. The *R*^2^ results from this process were aggregated into an average *R*^2^ value for each result at the different buffer sizes.

## Results

### Map of wind data sources

**Fig. 5:**
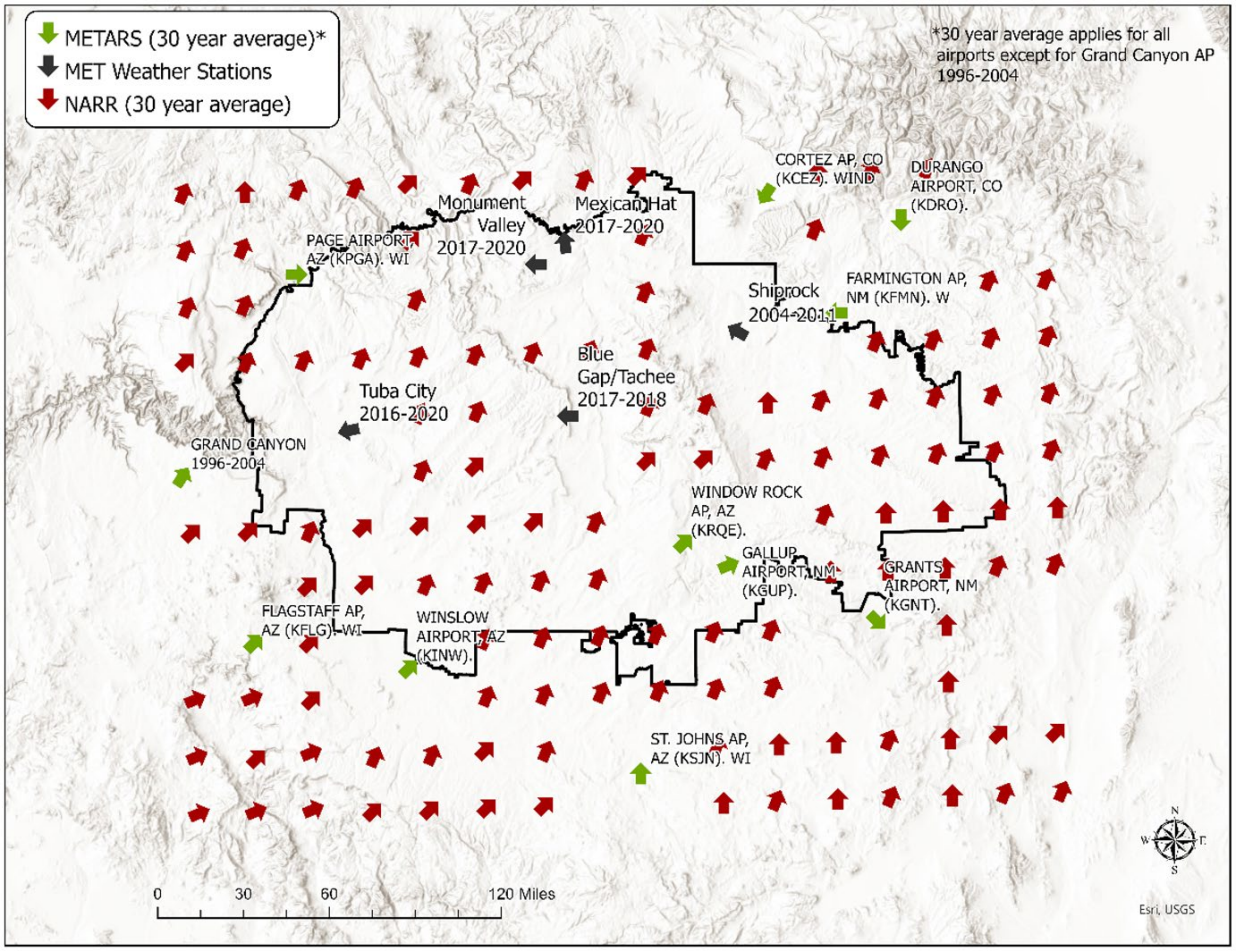
Wind direction data from different meterological data sources in the Navajo Nation

Figure 5 shows all three wind sources and wind directions based on each source as well their spatial coverage in the Navajo Nation. The NARR data covers the entire Navajo Nation, and the prevailing wind direction is Southwest. There are some discrepancies among the three sources. The METARs data agree with NARR data in all areas except the Northeastern area. The MET data are consistent with the METARs except in the Tuba city area, large disagreement is observed when compared against NARR.

### Modeling results

**Fig. 6:**
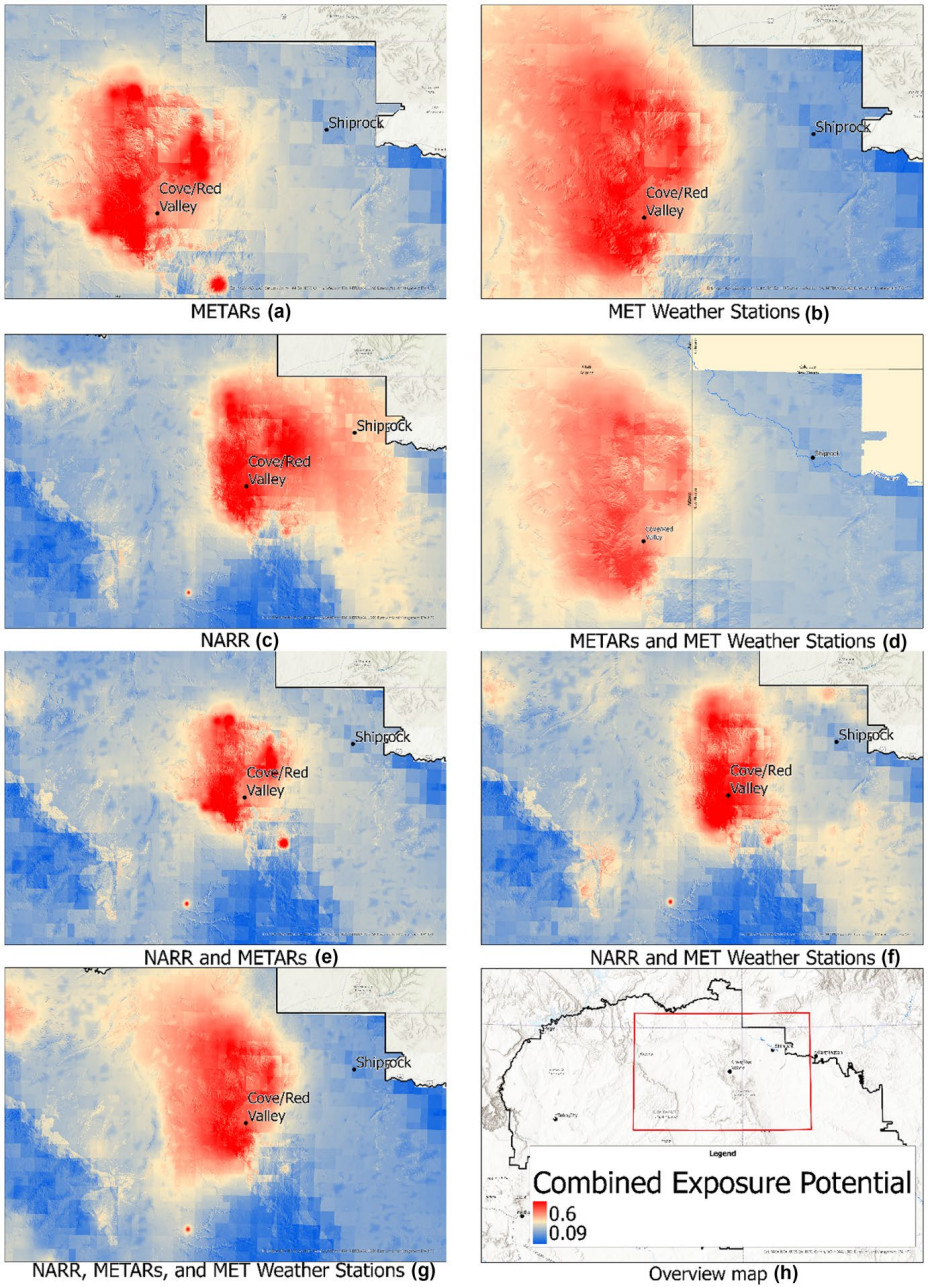
Potential environmental exposure to AUM predicted by the GIS-MCDA model with different version of meteorological data: METARs (**a**); MET stations (**b**); NARR (**c**); METARs and MET stations (**d**); NARR and METARs (**e**); NARR and MET stations (**f**); NARR, METARs, and MET stations (**g**); Overview of the Navajo Nation—with the area represented in the other figures outlined in red (**h**)

Figure 6 shows the potential AUM exposure in the Navajo Nation for each wind data source. Because of the clustered mine sites, the general areas with higher exposure potential remain the same throughout each version, but there are subtle differences. In versions of the model where NARR data are incorporated, it should be noted that the Southeastern and Northern clusters show higher potential exposure at a farther distance than other versions of the model. While the presence of high exposure potential proximal to the mine clusters is a consistent result of models with all met sources, the impact of data source can be seen to substantially influence less expected predictions in regions distal to the mines where variability can readily be seen.

### Statistical test results

**Fig. 7:**
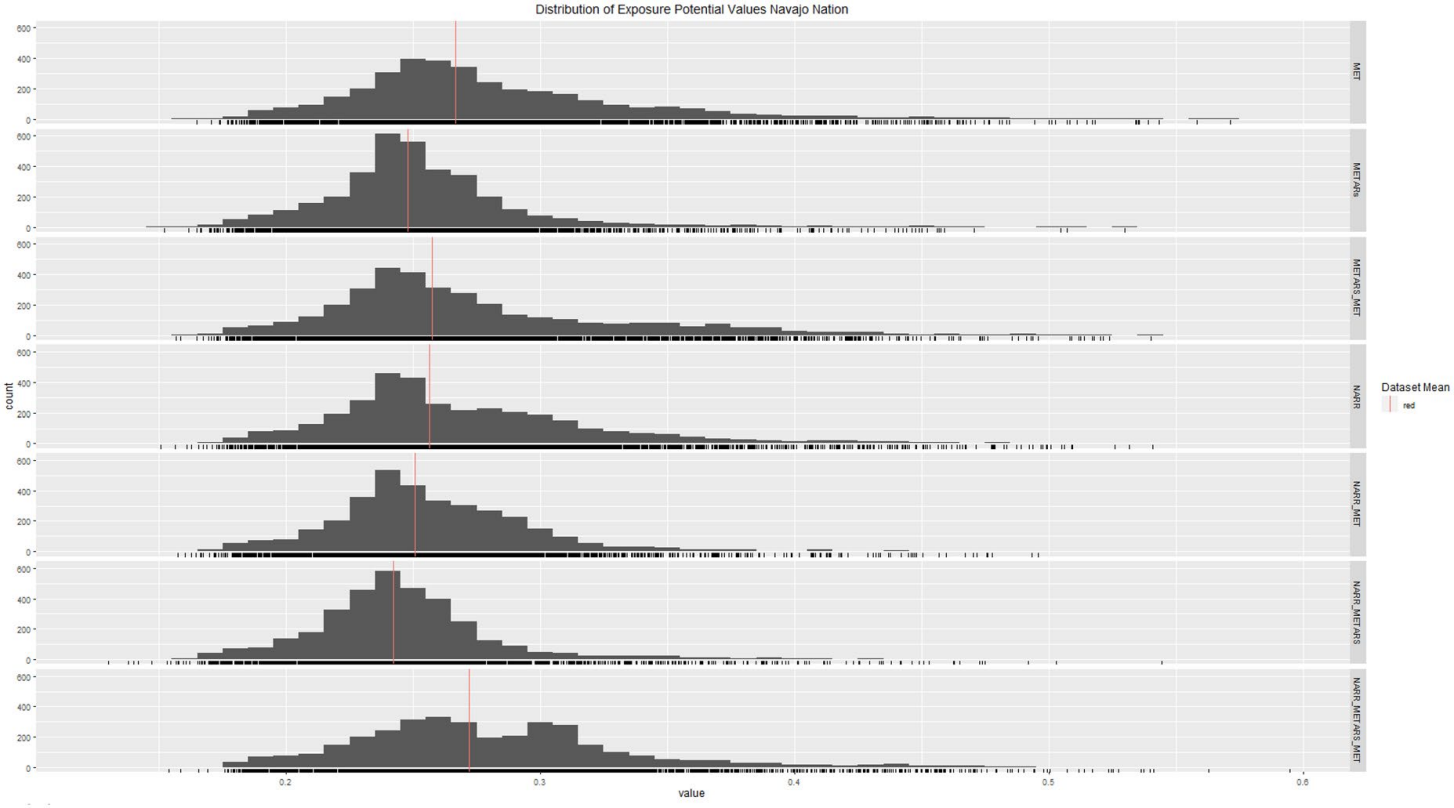
Frequency distribution of 500 random NURE samples from the modeling results derived from different meterological data sources (Note: the X axis represents the log (base2) uranium exposure potential value; the Y axis represent the frequency of each value; the red line represents the mean value for each result version

Figure 7 shows the distribution of 500 random points from each result version transformed with log2, along with the mean of each dataset. This study used an ANOVA test to compare between the different result versions for each study area. The ANOVA yielded a *p* value $< 2{e}^{-16}$, which is below the normal $\alpha$ level of 0.05, suggesting the means among the groups are not the same. This does suggest that there is a clear difference in the results depending on whether the model is inputting either local station data, modeled data, or some combination of the two.

### Validation results

**Fig. 8:**
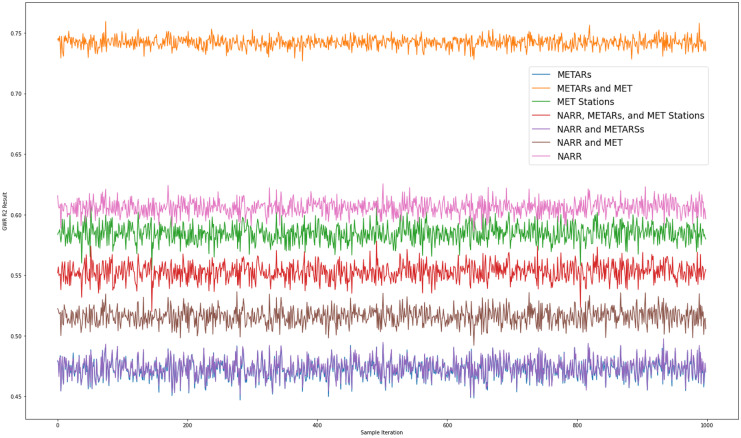
GWR validation results for each model version X-axis: sample number (0–999) Y-axis: GWR global *R*^2^ value

Table 4 shows the descriptive statistics *R*^2^ results from the validation for each version of MET data. The validation shows that the METARs and MET weather stations results consistently yielded the highest correlation with NURE data for the Navajo Nation when compared with other versions of MET data (Fig. 8). The *R*^2^ for NARR is the next highest according to the validation results. Local airport and the 5 MET stations alone produced lower *R*^*2*^ when compared with results generated from their combination or NARR.

**Table 4:** Descriptive statistics of *R*^2^ from the 999 validation tests

Statistic	METARs	NARR	MET stations	METARs and MET stations	NARR and METARs	NARR and MET stations	NARR, METARs, and MET stations
Mean	0.47	0.6	0.58	0.74	0.47	0.52	0.55
Standard deviation	0.008	0.007	0.007	0.005	0.008	0.007	0.007
Minimum	0.448	0.586	0.558	0.727	0.450	0.492	0.524
25% quartile	0.467	0.602	0.578	0.739	0.468	0.511	0.548
Median	0.472	0.606	0.585	0.742	0.473	0.516	0.553
75% quartile	0.478	0.610	0.590	0.745	0.479	0.521	0.558
Maximum	0.497	0.627	0.610	0.758	0.498	0.537	0.576

### K-fold CV results

**Fig. 9:**
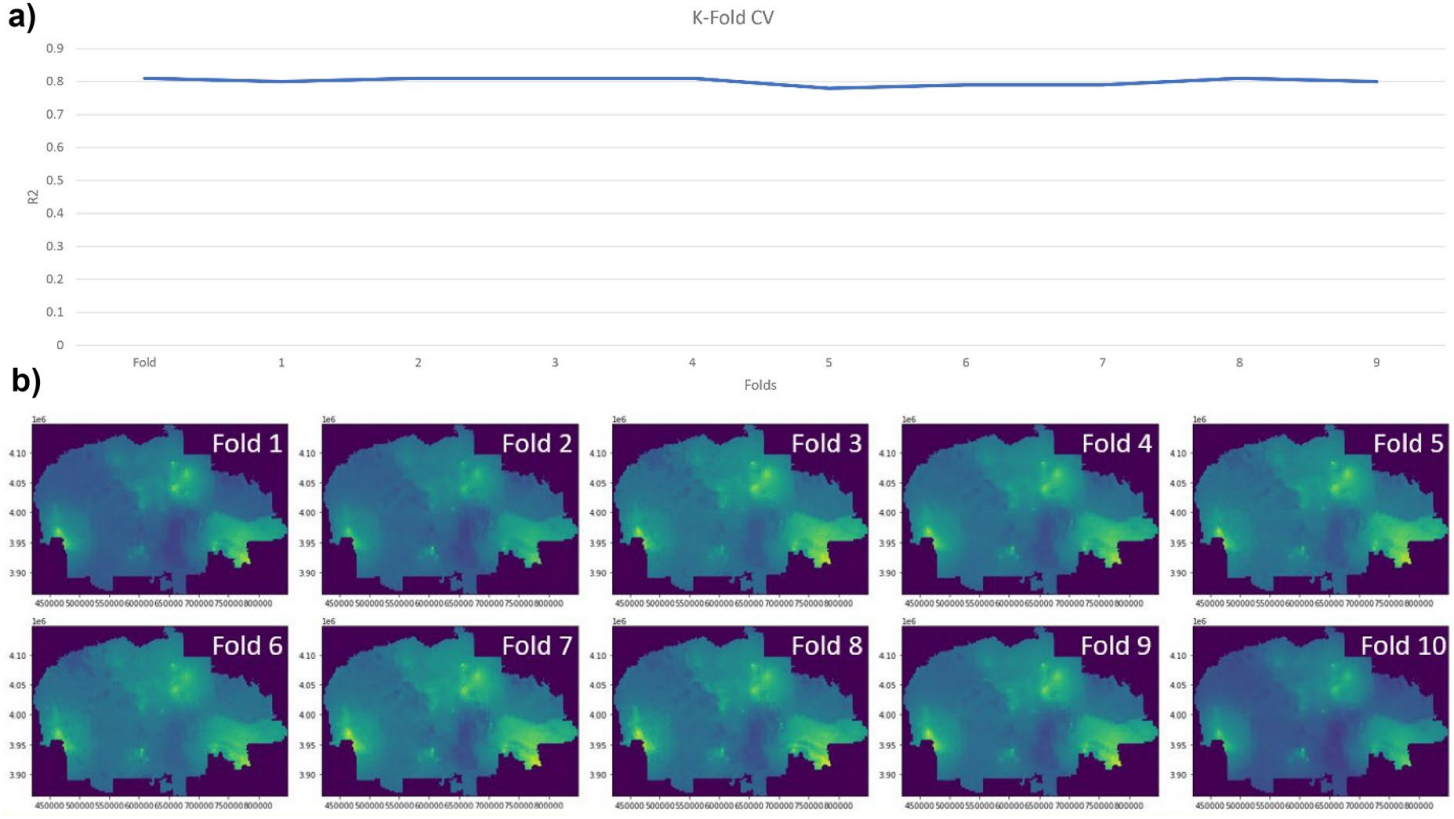
Predicted exposure surfaces across fold runs. Note high consistency in predicted surfaces

Plotting GWR *R*^2^ over the range of 10 test folds (with sample size *n* = 3500) indicates a highly stable model, with *R*^2^ values ranging from 0.79 to 0.81. The output exposure maps show slight variance but are overall highly consistent (Fig. 9).

### Scale variability analysis

**Fig. 10:**
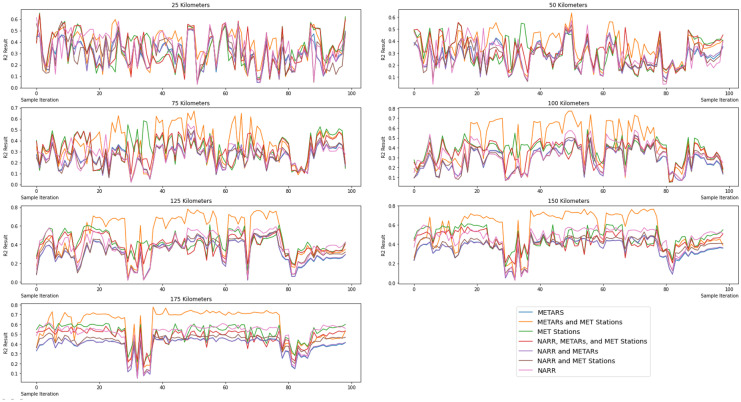
*R*^2^ value from the spatial scale tests using independent subset samples based on 99 iterations

Table 5 shows the average *R*^2^ results and the standard deviations of those results from the spatial scale test described in Sect. 2.9. The METARs and MET weather station version of the model had the highest average *R*^2^ value over all scales from 25 to 175 km. The standard deviations were between 0.1 and 0.14 for most of the result versions across various scales. The METARs and MET stations had higher standard deviations overall, with a range of 0.14–0.22. Figure 10 shows the *R*^2^ results from each sample point at the various buffer levels. These figures also showcase that in most of the iterations, the local airport and MET weather station version of the data shows much higher results than the other versions.

**Table 5:** Mean ± standard deviation of *R*^2^ value from scale test

Model	25 km	50 km	75 km	100 km	125 km	150 km	175 km
METARs	0.3 ± 0.12	0.27 ± 0.1	0.25 ± 0.1	0.28 ± 0.12	0.31 ± 0.13	0.36 ± 0.1	0.39 ± 0,1
MET weather stations	0.33 ± 0.15	0.31 ± 0.12	0.33 ± 0.13	0.37 ± 0.1	0.42 ± 0.1	0.47 ± 0.1	0.51 ± 0.1
NARR	0.35 ± 0.14	0.28 ± 0.12	0.28 ± 0.1	0.33 ± 0.14	0.39 ± 0.15	0.46 ± 0.13	0.5 ± 0.13
METARs and MET weather stations	0.36 ± 0.14	0.34 ± 0.13	0.36 ± 0.16	0.42 ± 0.22	0.49 ± 0.22	0.55 ± 0.19	0.6 ± 0.17
NARR and MET weather stations	0.31 ± 0.14	0.26 ± 0.1	0.25 ± 0.1	0.29 ± 0.12	0.34 ± 0.13	0.39 ± 0.1	0.43 ± 0.1
NARR and METARs	0.3 ± 0.12	0.27 ± 0.1	0.25 ± 0.1	0.28 ± 0.12	0.32 ± 0.13	0.36 ± 0.1	0.39 ± 0.1
NARR, METARs, and MET weather stations	0.33 ± 0.15	0.31 ± 0.12	0.32 ± 0.12	0.36 ± 0.1	0.4 ± 0.1	0.44 ± 0.1	0.47 ± 0.1

## Discussion

Previous work in MET data quality investigations in environmental exposure modeling is limited. There have been contradictory findings in the impact of MET data quality on modeling. For example, previous work focused on MET data other than wind found little to no significant differences between direct measurement and non-direct measurement-based MET data in the overall predictions of spatial models (Elaji & Ji, 2020; Klouče et al., 2015) although other research has concluded that non-direct measurement-based data were not accurate for short time scales (Futter, 2000). Other related research includes comparisons of the quality of MET data (Wilgan et al., 2015) and has shown significant differences in predicted weather scenarios derived from different input data. This is one of the first studies to examine the impact of various wind data sources as opposed to other MET variables such as rainfall (Elaji & Ji, 2020) and air quality (Futter, 2000). Several previous studies indicated that data from direct measurements with better spatial coverage were significantly more accurate than the interpolation based gridded data (Wilgan et al., 2015; Futter, 2000), which is corroborated by the present study. Most importantly, the present study adds to the literature through examining different wind data sources in a geospatial model for environmental exposure modeling.

The results presented here suggest that future studies should consider evaluating the impact of various MET data sources on the model results and consider limitations of available data in study design and interpretation. Researchers attempting to choose between MET sources should combine all available direct measurement sources and directly compare this source to either satellite or reanalysis data, leveraging processes/approaches (e.g., cross validation, 999 permutation test) to avoid model overfitting and to give a higher confidence or justification on findings about the most accurate data source. Future work could expand the assessment to other geographic areas (e.g., urban areas where MET data are ample) or other modeling efforts/contexts to see if similar findings can be produced. Although the current study is focused on wind data only, future studies that examine more than one type of MET data (e.g., wind and precipitation) are encouraged.

Based on the results of this study, future research in large-scale environmental modeling that rely on MET data should look to invest in MET stations to supplement the already available local data to enhance the spatial coverage. While the spatial distribution of these data will likely be uneven, which can introduce uncertainty into the modeling process, when used with an existing network of direct measurement sites it has proven to yield more accurate results than the gridded reanalysis NARR data. For future study of AUMs exposure potential in the Navajo Nation, this methodology might inform the decision-making process for the placement of future MET stations.

At all scales (25 – 175 km), the METARs together with MET weather station version returned the highest average *R*^2^ value. These findings from the scale tests further support the claim that utilizing local weather stations in places of interest with a better spatial coverage will yield more accurate results than using interpolated reanalysis wind data. However, we also observed that below 75 km, the *R*^2^ for the METARs and MET weather station model only slightly outperforms the others. This might indicate that the geospatial model in this case study is more suitable for larger scale studies (e.g., areas greater than 75 km). Another explanation could be related to the irregular spatial distribution of NURE data. Nevertheless, the randomized 999 permutation test mitigate the effect of irregular distribution of sample points. Future studies should test other models or use more evenly distributed validation data set if available.

### Strengths

This is the first study to investigate the impact of various wind data sources on geospatial modeling for environmental exposure purposes. This research clearly demonstrates that the type of input data used for modeling is critical to examine to understand and expose the impact of data quality on modeling. The results of this study show that using a well-distributed network of MET stations yield improved results over reanalysis-based MET data in a geospatial model. By utilizing a rigorous design of validation process, this study demonstrates the generalizability of the input data comparison using a geospatial model. 

Practically, this study directly informs further environmental health research in the Navajo Nation, including MET station placement, AUMs exposure modeling, and other environmental exposure modeling. For example, this study will identify candidate locations for new MET weather stations where no nearby stations exist, and locations where the model prediction was poorer across all wind data versions such as those without AUMs in the central Navajo Nation as shown in the present study. These candidate locations will be further reviewed and refined based on community input and local decision-making. With more data from these new MET station sites, future work will be able to utilize this more accurate data source to generate more accurate modeling prediction. Furthermore, this study suggests a broad context applicable to other Indigenous communities and other rural lands with limited MET data.

### Limitations

This study focused on the MET data quality in a geospatial model in a rural area with limited data sources. The generalizability to other settings cannot be determined without comparable work in other settings. Because of its rurality, the Navajo Nation has relatively fewer MET weather stations than an urban area would, limiting the spatial and temporal distributions of the sample network. Future studies should apply this process to an urban setting to determine if similar conclusions could be derived. The NURE dataset used to validate the data does not cover the entirety of the Navajo Nation, with a large portion of the Southwest study area having no NURE coverage. Another issue with this dataset is the date of collection, as the data was collected from 1975 to 1984, almost 40 years ago. Although there are more recent environmental data (e.g., soil data) collected by research teams working with the Navajo Nation, those data are only limited to small community level which could not be used together with NURE for the Navajo Nation wide validation for the geospatial model. The spatial distribution of the NURE data may have influenced the results of the permutation tests at smaller scales, leading to the METARs and MET station result only slightly outperforming the other versions. This may also have been a factor of the geospatial model, which was not originally intended for use at community-level scales (Lin et al., 2020). Future studies may need to focus on a smaller area where collecting a representative soil sample is feasible within the studies time frame and budget.

Regarding weight determination, there exist uncertainties inherent to ML. Importance scores given by RF are based on interpretations of the ML process and may not reflect an actual relationship (Breiman, 2001, 2017; Esri n.d.). Furthermore, RF has been shown to inflate relative importance for spatially autocorrelated variables (Strobl et al., 2008).A future study should test criteria layers for spatial autocorrelation and use transforms such as principal component analysis if significant spatial autocorrelation is detected (Maitra & Yan, 2008; Woldeyohannes, 2020). Additionally, this study used standardized settings for RF parameters, such as the number of decision trees, tree depth, leaf size, etc., when applying the RF algorithm. Future studies should conduct a sensitivity analysis to test RF weight determinations across different values/settings for algorithm parameters. Overfitting (Gavrilov et al., 2018) and ML bias caused by data leakage (Shim et al., 2021) can also be potential issues related to ML. Given the nature of this study, which required a large testing size for input into the GWR (*n* = 3500), meant that every test fold of the k-fold CV could not be completely independent of each other. Further tests are needed, using lower testing sizes, to see if the model is still stable at lower correlation values.

## Conclusion

This study set out to achieve 2 objectives: (1) Identify whether different meteorological data sources produce significantly different model results; and (2) determine the meteorological source that produces the most accurate result. The validation test, scale test, and the ANOVA results indicate that there is a significant difference in model performance when utilizing various forms of wind data. The *R*^2^ results from the validation tests indicate that direct measurement-based wind data with better spatial coverage or more meteorological stations yielded the most accurate exposure potential prediction, which is supported by the result presented here that METARs together with MET stations (both are direct measurement) with more stations outperform METARs or MET stations only predictions. The model results also suggest that the geospatial modeling in the present study using MET stations and METARs accurately represented small to medium scale spatial variations when compared with other meteorological data/data combinations, as evidenced by higher accuracy for scales greater than 75 km. When the reanalysis data was combined with direct measurements (i.e., NARR and MET stations; NARR and METARS; and NARR, METARs, and MET stations), the varying meteorological data sources conflicted with each other and produced lower *R*^2^ values.

## Data Availability

Data links and Code samples are available at the following figshare page:https://figshare.com/projects/Meteorological_Data_Source_Comparison_a_Case_Study_in_Geospatial_Modeling_of_Potential_Environmental_Exposure_to_Abandoned_Uranium_Mine_Sites_on_Navajo_Nation/150519
